# General and paediatric dentists’ knowledge, attitude and practises regarding the use of Silver Diammine Fluoride for the management of dental caries: a national survey in the Netherlands

**DOI:** 10.1186/s12903-022-02475-w

**Published:** 2022-11-01

**Authors:** Sofie C.H. Schroë, Clarissa C. Bonifacio, Josef J. Bruers, Nicola P. T. Innes, Daniela Hesse

**Affiliations:** 1 Department of Paediatric Dentistry, Academic Centre for Dentistry Amsterdam (ACTA), University of Amsterdam and Vrije Universiteit Amsterdam, Amsterdam, The Netherlands; 2 Department of Social Dentistry and Behavioural Sciences, Academic Centre for Dentistry Amsterdam (ACTA), University of Amsterdam and Vrije Universiteit Amsterdam, Amsterdam, The Netherlands; 3 Department of Research and Information, Royal Dutch Dental Association (KNMT), Utrecht, The Netherlands; 4 School of Dentistry, College of Biomedical & Life Sciences, Cardiff University, Cardiff, Wales

**Keywords:** Dental caries, Silver Diammine Fluoride, Silver Diamine Fluoride, Survey, Knowledge, Attitude, Barriers, Facilitators

## Abstract

**Background:**

Silver Diammine Fluoride (SDF) is a topical medication used to arrest cavitated carious lesions non-invasively. The primary aim was to investigate, and analyse the relationships between; knowledge, attitudes and practises (including barriers and facilitators) for SDF use in the management of dental caries by general dental practitioners (GDPs) and paediatric dentists (PDs) in the Netherlands. A secondary aim was to explore any differences in these, between these groups.

**Methods:**

A randomly selected sample of 600 Dutch GDPs (out of 9,502 respectively) and all 57 registered Dutch PDs were invited to participate in this cross-sectional survey, consisting of four sections: (1) participant characteristics, (2) knowledge (through responses to summative questions), (3) attitudes (through statement agreement using 5-point Likert scale), and (4) practises, use, barriers and facilitators (through multiple choice questions).

**Results:**

The response rates were: GDPs 23% (n = 140) and PDs 47% (n = 27). Knowledge: out of 15 questions to test understanding of SDF, the mean number of correct answers were GDPs 6.7; standard deviation (SD) 2.6 and PDs 7.4, SD 2.2 with no significant difference. The mean overall attitude score showed positive attitudes towards SDF use for both groups. Compared to GDPs, PDs were more likely to use SDF (p < 0.001) and expected to increase their use (p = 0.037). The main barrier for users was parental acceptance (47%) and for non-users it was lack of knowledge (60%). The main facilitator for both users and non-users was gaining knowledge through courses and workshops, followed by written information leaflets about SDF for parents.

**Conclusion:**

Less than half of the knowledge questions about SDF were answered correctly. Despite low knowledge, attitude towards SDF use was positive. Practitioners believed that its use would be facilitated by professionals having more accessible information and training and by the availability of parent information leaflets. Furthermore, SDF is used more frequently by PDs than GDPs.

## Background

The paradigm shift from traditional, towards minimally invasive dentistry, has resulted in increased interest in less traditional techniques for managing carious lesions [1]. One example is the use of Silver Diammine Fluoride (SDF), also known as Silver Diamine Fluoride, which is used to arrest dental caries in a non-invasive way and has recently grown in popularity. SDF’s mode of action is two-sided: the silver-ions are anti-bacterial and inhibit dentine collagen degradation, and the fluoride-ions (48.800ppm) enhance remineralisation [2, 3]. Together, in an alkaline solution, these components act synergistically to promote the arrest and remineralisation of carious lesions. SDF outperforms comparators like fluoride varnish in slowing progression of dentine lesions in the primary dentition [4]. Furthermore, it is a simple, safe, cheap, and effective treatment modality, and has been found to be useful for patients with high caries-risk and also for those who are unable to tolerate conventional invasive treatment [5, 6].

Despite these advantages, SDF’s most obvious disadvantage is its side-effect of permanent black staining of carious lesions [4]. The poor aesthetics associated with the black staining could lead to lower acceptability and satisfaction of parents, children or dental practitioners [7]. Investigations show that parental acceptance of SDF is higher when it is applied on posterior compared to anterior teeth with patients from lower socio-economic positions (SEP) and when children require more advanced methods of behaviour management [8]. Interestingly, other research has found that overall, the black stains were well accepted by both parents and patients, whereas this acceptance was low among dental professionals [7]. Therefore, dental professionals may not offer SDF as a treatment option if they assume that parents are unlikely to accept it because of concerns about the aesthetics [9].

Another factor that can influence SDF use is its relatively recent approval by the FDA in 2015 for use in the USA. As a fairly new material, knowledge about it amongst dental practitioners might be low. Indeed, a survey of Brazilian dental practitioners reported the main barrier to the use of SDF to be lack of knowledge and in Saudi Arabia and the USA, a positive relationship between knowledge, attitude and the use of SDF was found [5, 10, 11].

Since its approval by the FDA, SDF has also become available in dental practice in European countries. However, to our knowledge, no survey regarding this subject has been performed in Europe. In the Netherlands, the caries prevalence in 5-year-old children has decreased considerably. However, socio-economical inequalities are still present and are responsible for caries disproportionally affecting the disadvantaged in the population. The latest data from 2017 show that 25% of 5-year-old children had experience of dental caries [12]. From those, the low SEP group had a prevalence of 29% compared to 19% from the high SEP group. The prevalence reached 74% when the children’s mothers had a migration background, compared to 22% when their mothers were born in NL. Despite a system with full paediatric dental coverage, a proportion of these carious lesions is left untreated and the prevalence of caries in 11-year-olds in the low-SEP group was 43%, in 2017 [13]. As caries disproportionally affects disadvantaged groups, SDF could be considered a cost-effective treatment option to arrest caries lesions in children [13]. The two groups that are most likely to be using SDF in their practice, in the Netherlands, are general dental practitioners (GDPs) and paediatric dentists (PDs). By investigating the knowledge of a topic, researchers can get an overview of understanding around a specific subject within a population. When the attitudes are investigated, the feelings and preconceived ideas that a community has regarding a particular topic are studied, while investigating the practises concerns the ways in which they demonstrate both their knowledge and attitudes through their actions [14]. Therefore, surveying the two groups of dentists about their knowledge, attitude and practises regarding SDF use for the management of dental caries will give insight into their awareness about this topic and whether more education or training is needed to improve attitudes towards its use [15].

This study aimed to: (1) assess knowledge, attitudes and practises regarding SDF among GDPs and PDs in the Netherlands; (2) investigate the relationships between knowledge, attitudes and practises; and (3) explore differences between the two groups of oral health care workers.

## Methods

This survey is reported according to the Strengthening the Reporting of Observational Studies in Epidemiology (STROBE) guidelines [16]. The study was approved by the Research Ethics Committee from the Academic Centre for Dentistry in Amsterdam (ACTA), the Netherlands (#2,020,265).

### Sample size calculation

Sample size calculation for the GDP-group was performed using OpenEpi (Version 3, open-source calculator-Powermean). It was based on a rate of 13% general use of SDF (among GDPs and PDs) [10]. Taking into account that there are currently around 9,502 registered GDPs in the Netherlands, the estimated sample size was 123 with a confidence level of 90%. Since the average response rate of Dutch GDPs to written surveys is reported to be around 30% [17–19], we increased the sample size to 70%, resulting in 600 invitations to members. For the PD-group, all 57 registered pediatric dentists were invited to participate into this research.

### Study population & recruitment of participants

The study involved two groups: (1) GDP-group: a random sample of registered GDPs with a valid home and working address in the Netherlands aged 67 years or younger; (2) PD-group: all PDs registered in the Dutch Society of Paediatric Dentists.

The participants were contacted via letter, which contained a written questionnaire. Additionally, the letter contained a link to the online version of the questionnaire, giving the respondents the option of responding on paper or online. This letter included information about the purpose of the research, the relevance of the study, the estimated amount of time to complete the questionnaire, and that participation was voluntary. Responding to the survey was considered as implicit informed consent; therefore, no explicit consent to participate in this research was required. A reminder email was sent to non-responders after one and again after two months. Sending the questionnaires was commissioned by the Royal Dutch Association for Dentistry (KNMT) and carried out by a third-party research agency. KNMT manages a reliable database of all 9,502 qualified dentists aged 67 years or younger who live and/or work in the Netherlands. For research upon request, and subject to security and privacy protection conditions a random sample can be made available from this database. So, a random sample of 600 GDPs was drawn and mailing and email addresses were provided. These addresses of the PDs are openly available on the website of the Dutch Association for Paediatric Dentistry.

### Data collection & questionnaire

The third-party research agency sent the questionnaire to the GDPs and to the PDs, giving them each a unique number and they also processed the returned questionnaires. The agency was able to identify non-responders and therefore send the follow-up emails. Data collection was carried out between December 2020 and February 2021. The online survey was set up using the platform LimeSurvey (LimeSurvey GmbH, Hamburg). The recruitment of participants was according to the General Data Protection Regulations.

We used a 20-item questionnaire, based on questions used in two previous studies that evaluated the perceptions of dental hygienists regarding the treatment of dental caries using SDF and the education, knowledge, attitudes and professional behaviour of paediatric dentists regarding the use of SDF [5, 20]. The survey was pre-tested on a convenience sample, consisting of 10 dentists who work at the Department of Paediatric Dentistry at the Academic Centre of Dentistry in Amsterdam (ACTA) to assess acceptability and feasibility. Survey modifications were made based on the feedback.

The questionnaire consisted of four parts: (‘1) demographic details and information regarding the clinical experience of the respondents; (2) respondents’ knowledge about the use of SDF (by using multiple choice questions, where the possible answers were ‘correct’, ‘incorrect’ or ‘I don’t know’); (3) respondents’ attitudes towards the use of SDF (by scoring statements based on practitioner’s level of agreement using the 5-point Likert scale) and how acceptable they found SDF treatment’s aesthetics; and (4) respondents’ own use of SDF and exploration of potential barriers and facilitators for the use of SDF (by selecting the barriers/facilitators that were listed or by adding a new one to the existing list). The complete survey is presented in Additional file 1.

### Statistical analysis

Statistical analysis was performed with IBM SPSS Statistics version 22.0 (Statistical Package for the Social Sciences; SPSS, Chicago, IL, USA). Descriptive statistics, such as frequency distributions, percentages and means were computed to provide an overview of the responses. To test knowledge, scores from different items were added up; hence, knowledge has been considered both within sections and overall. To report the frequency of the barriers and facilitators, 3 groups were presented: the top 1/3, middle 1/3 and lower 1/3. To test the coherence between the statements with the 5-point Likert scale, Cronbach’s alpha was computed. To test for correlations, chi-square test, independent sample t-tests and Pearson correlations were carried out. Imputation of missing data was not carried out. Furthermore, we performed a *post hoc* test to verify the power of the sample regarding knowledge, attitude and use. It resulted in a power of 35% regarding both knowledge and attitude, and a power of 100% regarding SDF use. For all statistical tests, the significance level was taken as 0.050.

## Results

### Survey response rates

There were responses from 167 dentists (overall response rate 25%), of whom 140 were GDPs (23% of invited GDPs) and 27 PDs (47% of invited PDs). In the GDP-group, 32 respondents were not actively treating patients, so they were excluded from the survey, resulting in 106 GDPs (18%) included in the analysis.

### Participant characteristics

Table 1 shows participant characteristics by GDP-group and PD-group.

There were more females in the PD-group (p = 0.004), they were older (p < 0.001) and consequently had more years of clinical experience (p = 0.009) than those in the GDP-group. As expected, more PDs reported working in a referral practice for children (p < 0.001) and treated more patients under 13 years of age (p < 0.001).

More than half of the GDPs (52%) indicated that they were familiar with SDF, while all PDs (100%) were familiar with it (p < 0.001). The term ‘not being familiar’ means that the respondent had never heard of SDF or did not know what it was. Consequently, they had no knowledge about it, nor an opinion and were excluded from further analysis. Therefore, responses for the 50 GDPs who were not familiar with SDF were not considered in the remaining analyses, resulting in data for 56 GDPs. There were no significant differences regarding age, gender, working place and proportion of children that are treated under the age of 13 years between practitioners who considered themselves familiar and those not familiar with SDF (p > 0.050).

**Table 1:** Participant characteristics (n = 133)

Participant characteristics		GDP-group (n = 106) n (%)	PD-group (n = 27) n (%)	Test (Chi-square)
Gender	Male Female	49 (46) 57 (54)	4 (15) 23 (85)	p = 0.004*
Age (years)	< 35 35–50 > 51	43 (40) 27 (25) 36 (34)	1 (4) 19 (70) 7 (26)	p < 0.001*
Years of experience	< 1 1–5 6–10 > 10	1 (1) 32 (30) 12 (11) 61 (57)	0 1 (4) 1(4) 25 (92)	p = 0.009*
Working place	GP GP + other RPC RPC + others GP + RPC Other	95 (90) 6 (6) 0 0 4 (4) 1 (1)	0 2 (7) 14 (52) 2 (7) 7 (26) 2 (7)	
Children treated under the age of 13y	0 < 25% 25–50% 51–75% > 75% 100%	3 (3) 93 (87) 9 (8) 1 (1) 0 0	1 (4) 2 (7) 2 (7) 2 (7) 11 (41) 8 (30)	p < 0.001*
Familiarity with SDF	Yes No	56 (52) 50 (48)**	27 (100) 0	p < 0.001*

n = number of respondents; GDPs = General dental practitioners; PDs = Paediatric dentists; GP = General practice; RPC = Referral practice for children; Other = Centre for Special Care/University/Hospital/Referral practice for other patient groups than children; * denotes statistically significant difference between PDs and GDPs **not considered in further analyses

### Knowledge

Table 2 depicts the results related to the knowledge about SDF, assessed based on responses to 15 questions. In both groups less than half of the questions were answered correctly, with no difference between GDP-group and PD-group for overall knowledge (p = 0.260). There were no statistically significant differences between the groups for knowledge related to clinical indications (tooth or patient) and toxicity. However, a significant difference among the groups was noted regarding the mode of action of SDF, as more PDs responded with correct answers (p = 0.012). Furthermore, about 84% of the respondents knew that SDF was indicated for the management of active dentine carious lesions in the primary dentition (PD-group = 78%; GDP-group = 87%).

**Table 2:** SDF-related knowledge – Number of items scored correctly

Knowledge	GDP-group (n = 56)	PD-group (n = 27)	
	Mean number of items scored correctly (SD)		Independent samples T-test
Tooth indication (6 items)	3.1 (1.4)	3.3 (1.1)	p = 0.730
Patient indication (7 items)	3.8 (1.9)	3.9 (1.6)	p = 1.000
Mode of action (1 item)	0.3 (0.4)	0.6 (0.5)	p = 0.012*
Toxicity (1 item)	0.1 (0.4)	0.2 (0.4)	p = 0.310
Overall knowledge (15 items)	6.7 (2.7)	7.4 (2.2)	p = 0.260

n = number of respondents; GDPs = General dental practitioners; PDs = Paediatric dentists; SD = standard deviation; * denotes statistically significant difference

The most frequently mentioned source of knowledge differed between the groups; for PDs it was congresses/workshops and for GDPs, dental magazines/articles (Table 3).

**Table 3:** Source of information about SDF

Source of information (multiple answers possible)	GDP-group (n = 56) n (%)	PD-group (n = 27) n (%)	Test Chi square
Basic dental education	11 (20)	0 (0)	p = 0.013*
Postgraduate course	1 (2)	6 (22)	p = 0.002*
Congress/workshops	14 (25)	23 (85)	p < 0.001*
Dental magazine/article	31 (55)	19 (70)	p = 0.250
Colleague	16 (29)	15 (55)	p = 0.017*

*n = number of respondents; GDPs = General dental practitioners; PDs = Paediatric dentists*

### Attitude

**Fig. 1:**
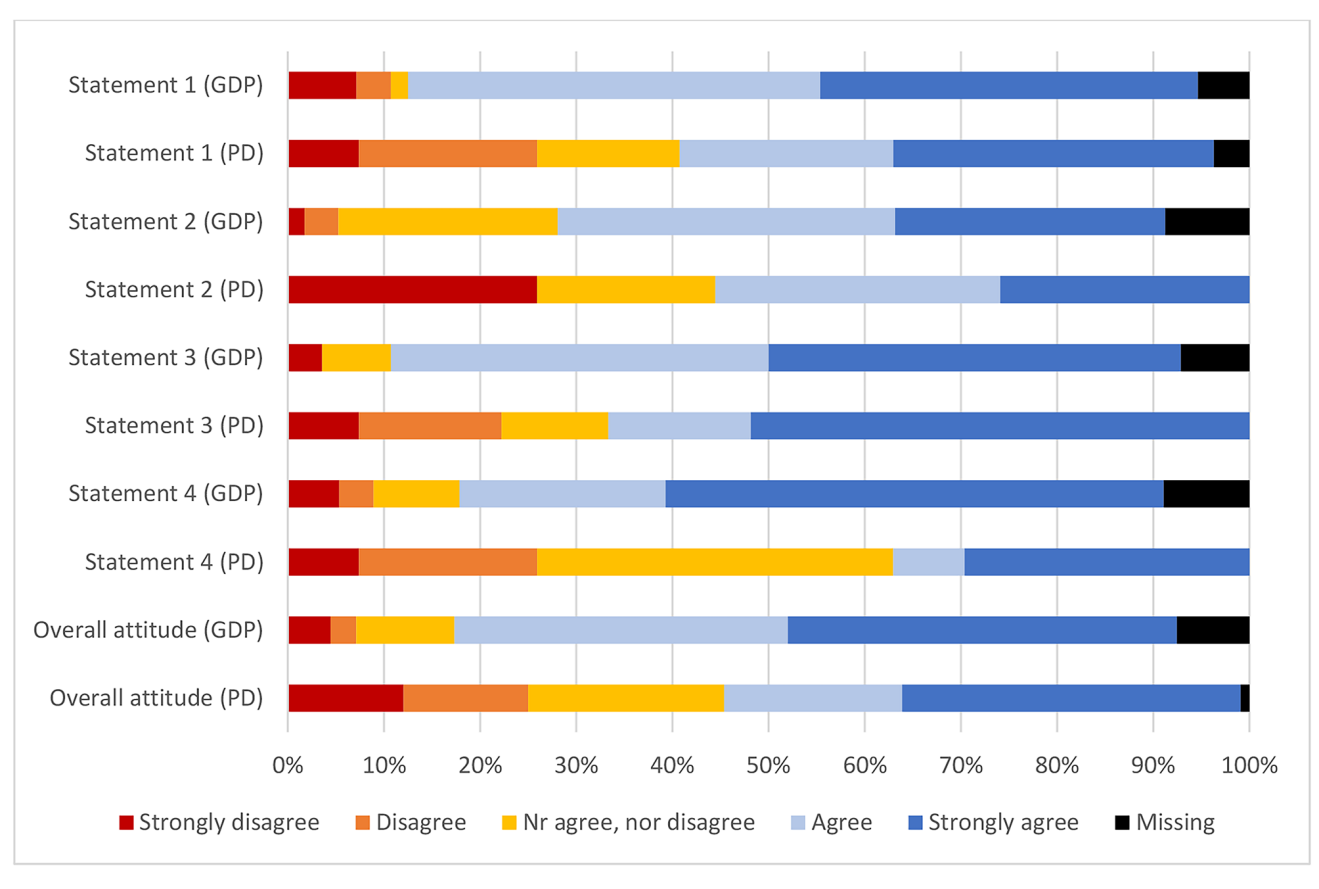
GDPs’ (n = 56) and PDs’ (n = 27) attitudes towards the use of SDF (absolute numbers are displayed in the bars) and detailed text for each statement is given below the figure. (*Statement 1: “For the treatment of caries, SDF should also be used by the dentist-general practitioner, not just a specialised dentist I think SDF should be used by GDP, not only by PD”, Statement 2: “The application of SDF is child-friendly”, Statement 3: “I am considering treatment with SDF in situations where conventional treatment is not (yet) possible”, Statement 4: “If possible, I would prefer treatment with SDF to treatment under general anaesthesia for my patients”*)

Figure 1 shows respondents’ attitudes towards SDF, which was assessed through four statements using a 5-point Likert scale. Cronbach’s alpha between the four statements was 0.820, showing reliable coherence between them. The sum score of these statements indicated the overall attitude of the respondents, that is, the higher the score, the more positive the attitude of dentists towards the use of SDF. It turned out that a more positive attitude was perceived among the GDP-group (GPD-group: mean score = 14.35 versus PD-group: mean score = 16.65; p = 0.012).

**Fig. 2:**
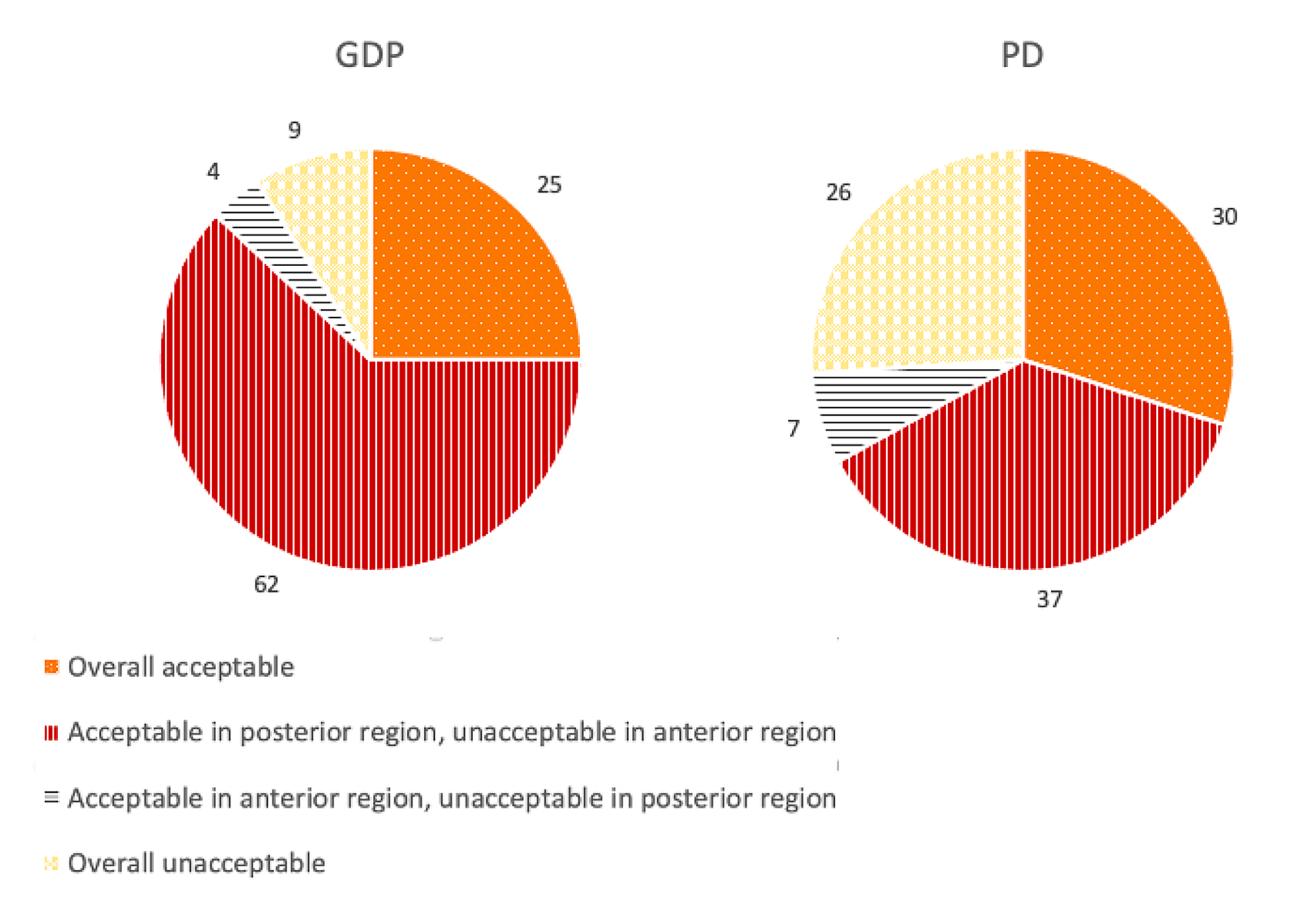
Aesthetic acceptability of SDF treatment among GDPs (n = 56) and PDs (n = 27) in percentages

Another statement was about the aesthetic acceptability of SDF treatment, which can be seen in Fig. 2. Considering the aesthetics and discolouration, there was no relevant evidence of a difference in reaction to this statement between the groups (p = 0.089).

### Practises

**Fig. 3:**
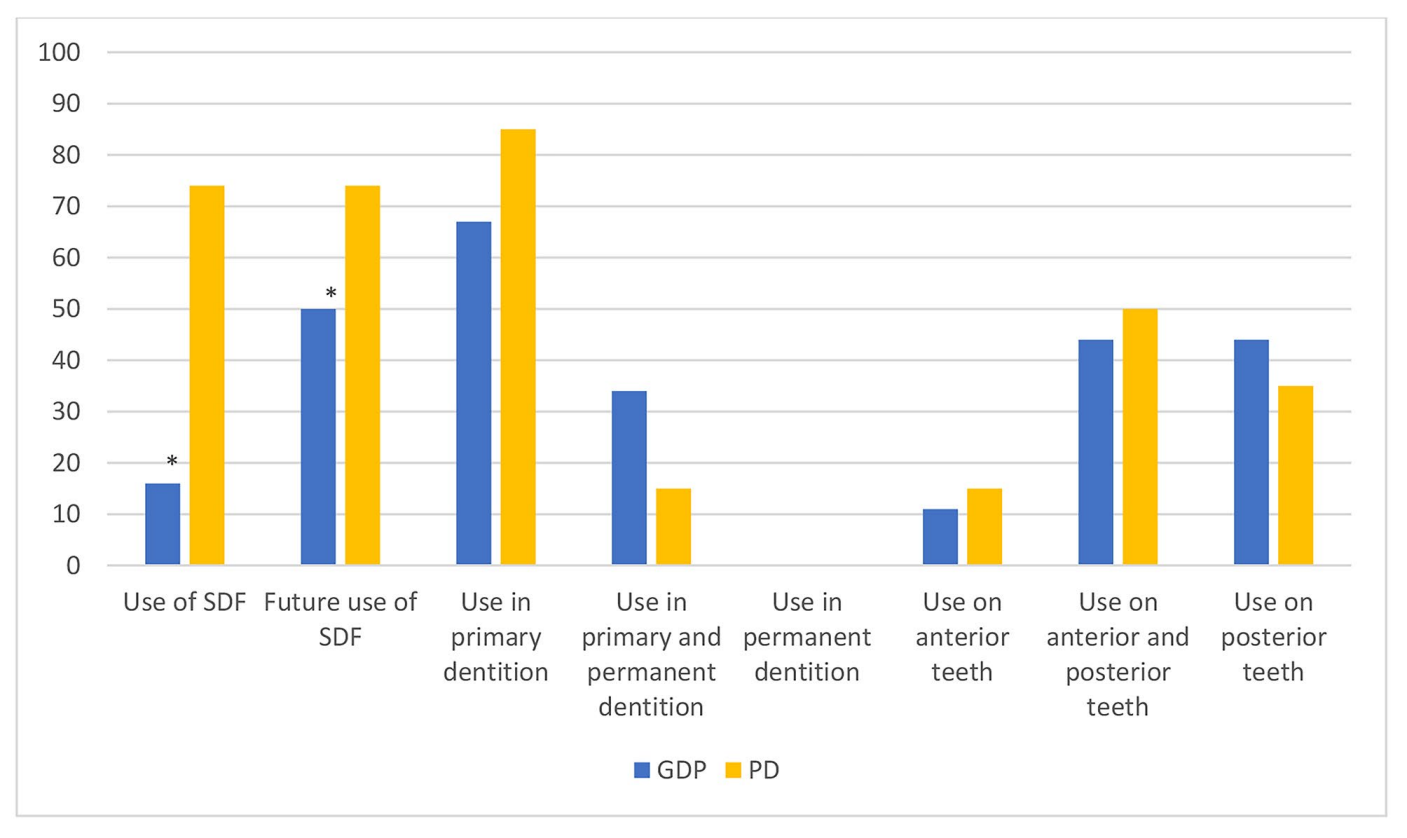
Percentage of respondents that used SDF, specified by dentition and location. (* denotes statistically significant difference)

Figure 3 shows the percentages of the respondents current, planned and future use of SDF for specific situations. PDs reported using SDF significantly more often than GDPs (PD-group:74%; GDP-group:16%; p < 0.001). In addition, PDs were more positive about their future use of SDF (PD-group:74%; GDP-group:50%; p = 0.037). Both groups most commonly used SDF alone in the primary dentition, although some dentists reported using it in the permanent dentition as well. Most dental practitioners who used SDF, had used it on both the anterior and posterior teeth of their patients. There were no significant differences about the use of SDF regarding the dentition and location between the two groups (p = 0.260 and p = 0.880).

Regarding the barriers to, and facilitators of, use of SDF. For users, the most frequently reported barriers were parental acceptance, not knowing the billing code and the risk of staining clothing/surfaces. For non-users, the most reported barriers were inadequate knowledge about SDF, parental acceptance and not knowing the legislation around SDF (Table 4). In both groups, the most frequently reported facilitator was improved knowledge through courses/training, followed by a parent information leaflet about SDF to give to them. In addition, almost half of the non-users wanted to find out about the experiences of colleagues to help them use SDF (Table 4). Table 4 shows the number of reported barriers and facilitators.

**Table 4:**
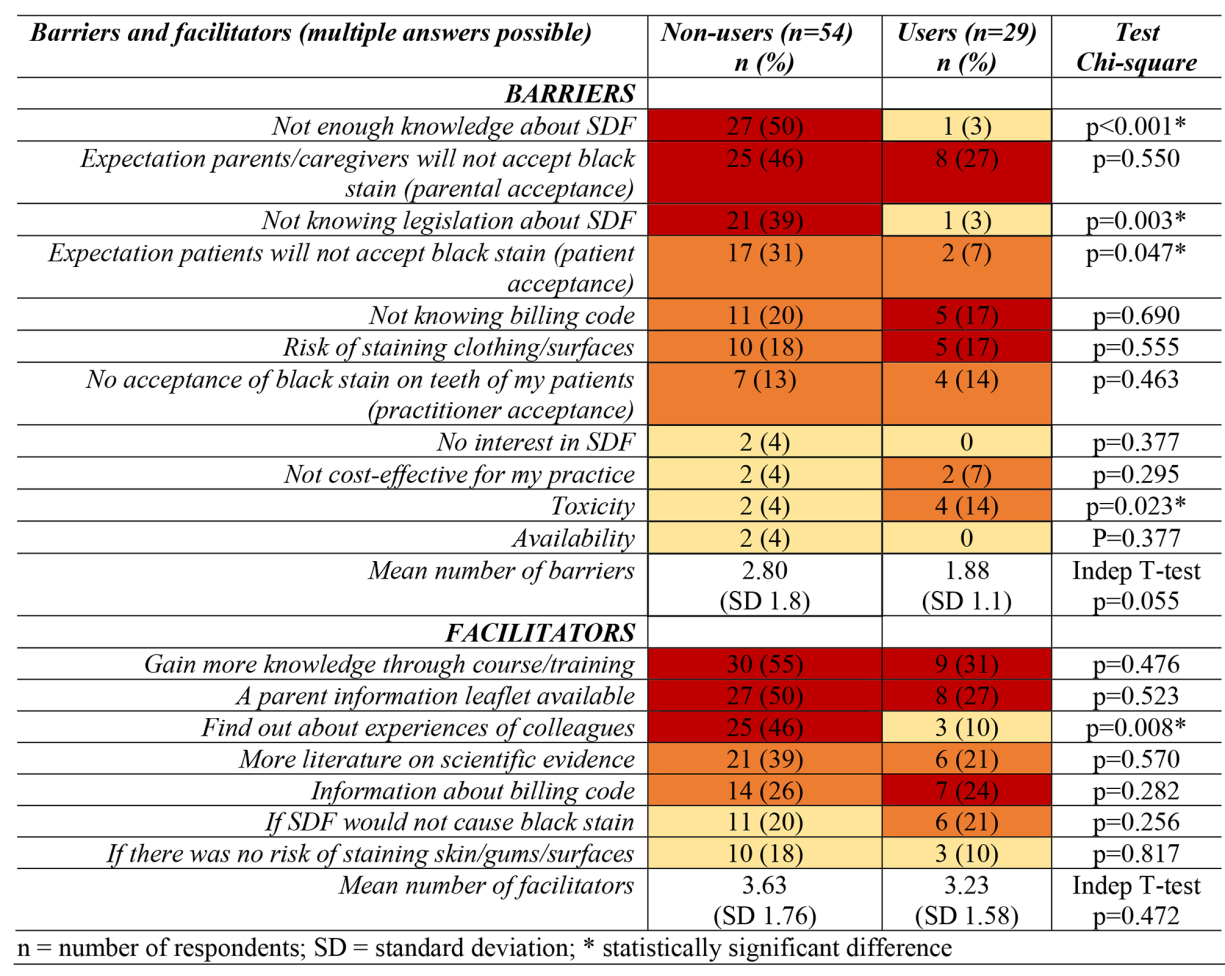
Barriers and facilitators to the use of SDF amongst users and non-users of SDF (barriers and facilitators have each been differentiated into three groups; the top 1/3 of responses is colored red, the middle 1/3 is orange and the bottom 1/3 is colored yellow)

### Relationships

Considering the data for both groups together, having more knowledge was associated with a positive attitude (p < 0.001) and greater use of SDF (p = 0.039). Furthermore, SDF use was associated with being a paediatric dentist (p < 0.001), working at a referral practice for children (p < 0.001) and treating children under the age of 13 years (p < 0.001).

## Discussion

This survey indicated that knowledge about SDF among dental practitioners in the Netherlands was low, but their attitude was positive. The low level of knowledge may be due to the fact that SDF has only recently been added to the clinical guidelines for dental practice in the Netherlands and to the dental curricula at universities [21]. This also explains the results related to the sources of knowledge, as basic dental education and post-graduate programs only play a small role in the acquisition of knowledge about SDF. Another explanation is that Dutch GDPs may be reluctant to switch to minimally invasive dentistry [19]. Furthermore, it is a challenge to transfer scientific evidence into practise and it might take several decades to fully adapt new clinical practises in dentistry [22–24]. Also, dentists who felt they had more knowledge about SDF were more likely to have a positive attitude towards SDF and use it more. These findings are in line with other studies [5, 11], indicating that further education about SDF may help increase its use by dental practitioners.’

The respondents’ attitudes were overall positive, indicating they would be willing to use SDF, but their lack of knowledge was a barrier. One could argue that the acquisition of more information about SDF would be enough to increase its use; however, it has long been accepted that it takes more than knowledge to change clinical behaviour [24]. Other than having the skills and knowledge; motivation and opportunity play important roles in accomplishing behaviour change. There must be a strong intention to perform the new behaviour and there should be no external factors, such as cultural, behavioural or socio-economic aspects, that prevent it being performed. According to our research, the intention to use SDF is present among the respondents, but, as might be expected, socio-cultural factors involved in the decision making could prevent dental practitioners from using it. Raising awareness and making it ‘normal practice’ to apply SDF in dental practice could increase its use. Furthermore, exploring whether cultural, behavioural or socio-economic aspects of the population influences the attitude and practises towards the use of SDF could be interesting and aid the development of actions to overcome the possible barriers for its use.

Most respondents in both groups would consider using SDF when conventional treatment was not possible and most of them agreed that SDF should be used in general dental practice, not only in referral practises. Surprisingly, the statement that SDF treatment was considered to be child-friendly had the lowest agreement among the respondents. Despite the advantages about the SDF use, such as no need for use of local anaesthesia, no caries removal, short chair time and being painless, some disadvantages are also reported [25]. For instance, SDF has a metallic taste, if in contact with the oral mucosa, a mildly painful white lesion can appear on this tissue it and the permanent black stain on the carious lesion could be considered not to be child-friendly. The majority of GDPs agreed that they would use SDF to avoid general anaesthesia, whereas only few of PDs agreed with this. This could be because most PDs in the Netherlands have access to facilities to allow treatment of their patients under general anaesthesia when necessary, so they may make more use of those facilities compared to GDPs.

SDF did not appear to be widely used by dental practitioners in the Netherlands with only around a third of respondents having used it in their practise. PDs used SDF more than GDPs, but neither used it on a daily basis. The non-use of SDF might be related to the frequently reported barrier of parental acceptance. This was also recently investigated by Magno et al. in 2019 and Seifo et al. in 2020 [7, 9]. Both studies found that dental professionals assumed that parents would not accept the black stain on their children’s teeth because of the risk for their child of being bullied at school or that others may judge them as neglecting their child’s oral health. A recent systematic review found that parental acceptance of SDF was better for posterior teeth than anterior teeth, and that the acceptance increased in time during the follow-up visits [26]. Another important finding from this review was that parents who had received instructions regarding indications and SDF use, showed less resistance to SDF compared to the parents who did not receive this information. This provides further support for the use of information leaflets to introduce SDF to the parents and patients. An example of this information leaflet can be downloaded on the website of the British Society of Paediatric Dentistry [27]. The barriers and facilitators related to SDF use in our study were in line with the ones reported by a previous investigation conducted in Scotland [9]. The barrier ‘availability’ was added into this research, as some participants don’t know how to purchase SDF for their practice.

In the Netherlands, the average response rate of GDPs to written surveys is reported to be around 30% [17–19]. Multi-approaches have been suggested in order to minimize non-response, measurement errors, and costs. A previous investigation carried out in the USA showed that a sequential web-paper-mail survey raised response and improved coverage for the general population [28]. In an attempt to increase the response rate in this research, we approached eligible respondents by sending a letter, which contained a written questionnaire together with a link to the online version of the questionnaire, giving the respondents the option of responding on paper or online. Despite these efforts, a response rate of 25% was reached in our study, which is to be expected with postal surveys [29].

An evaluation of the psychometric properties of the questionnaire used in this study was not carried out; however, we used the same kind of questions that were used in previous studies to test the different constructs [5, 22]. Nevertheless, we showed by means of a fairly high value of the Cronbach’s alpha that the statements regarding the use of SDF were interrelated, indicating a general attitude about it.

Low response rates are a potential source of bias, since it could result in misleading findings and only be representative of those who are more interested in the investigated topic and were more positive or negative in their responses. That may also explain the difference in response rate among GDPs and PDs, since it is possible that the non-responders were unfamiliar with SDF, which led to non-participation. That fact could rise the discussion regarding the internal validity of the present study; but the threats related to the internal validity are especially relevant in studies that try to establish a causal relationship between an independent and a dependent variable within the context of a particular research, and are, therefore, less relevant in most observational or descriptive studies [30]. So, this research may have overestimated the knowledge, attitude, and practises regarding the use of SDF among Dutch dental practitioners. Also, the statistical power regarding knowledge and attitude was only 35%, so care must be taken in drawing conclusions about these constructs. Nevertheless, the results provide some initial information on the knowledge, attitudes and practises about SDF use among dental practitioners in the Netherlands, which will be of great value when further planning educational strategies regarding SDF use in paediatric dentistry.

The aim of this research was to investigate the knowledge, attitudes and practices related to SDF use for the management of dental caries among dental practitioners who are currently active in carrying out patient treatment. The Dutch national retirement age in 2021 was 66 years and 4 months; therefore, the questionnaires were distributed among practitioners aged up to 67 years [31]. By doing this we aimed to reduce the number of professionals who were no longer practicing dentistry.

The amount of missing data was very low (< 1%) and imputation of missing data was not carried out. This meant that missing data, which can be a pervasive source of bias, did not influence the results. On the other hand, rejecting incomplete data can jeopardize inclusion in statistical analysis and reduce statistical power [32].

The prevalence of caries among Dutch children has considerably decreased in the past years [12]. In a previous investigation carried out to provide insight into caries prevention policies and services among children across EU member states, the Netherlands received high scores regarding access to dental caries screening and assessment of preventive services, data registration during the dental screening process, and financial aspects of preventive and operative treatments [32]. Therefore, the results of our investigation may not be representative of all European nations. Still, the caries experience among Dutch children follows the phenomenon of polarization, where affected children often present with a high number of carious teeth with deep lesions. The application of SDF is a simple, safe, cheap, and effective approach for arresting caries lesions in children, and has been found to be useful for treating patients presenting several caries lesions, or those considered as high caries-risk or children unable to tolerate conventional invasive treatment [5, 6]. For this reason, investigating the knowledge, attitudes and practises related to SDF use for the management of dental caries by GDPs and PDs in the Netherlands is important and the results found in our investigation may help in planning educational programs to increase the use of SDF in this population. Furthermore, future research among other European dentists could give more insight into the investigated topics of this research in Western countries. Incorporation of subjects like clinical efficacy, availability of the product in their country or practice, barriers for reimbursement and costs of the treatment/material could be included in future research. Nevertheless, qualitative research among dental practitioners could be carried out in order to understand the barriers to SDF use.

There are some recommendations that can be made from this survey. As well as education being demonstrated as the most important facilitator, we also noted that some respondents commented in the survey that they want to undertake a course or training. So, this research has created awareness of the need for development and availability of courses/training about SDF in the Netherlands. During this training, information about billing codes, legislation, and availability should be given as well as the clinical information including indications, contra-indications and procedure for using SDF. In addition, parent and child information about SDF should be available, as this was the second most commonly reported facilitating factor.

## Conclusion

Knowledge of SDF among Dutch dental practitioners was low, but their attitude towards its use was positive. Having high levels of knowledge was associated with a more positive attitude and greater use of SDF. The most commonly reported barrier to its use was the lack of knowledge, followed by dental professionals’ concern over parental acceptance of the black staining.

## Data Availability

All data generated or analyzed during this study are included in this published article.

## List of abbreviations

SDF: silver diammine fluoride.
GDP: general dental practitioner.
PD: paediatric dentist.
FDA: Food and Drug Administration.
USA: United States of America.
KNMT: Koninklijke Nederlandse Maatschappij voor Tandheelkunde.
GP: general practice.
RPC: referral practice for children.
SEP: socio-economic position.
